# Plasma Proteomic Analysis Reveals Complement System Changes in Irradiated Female BALB/c Mice during Mammary Carcinogenesis

**DOI:** 10.1158/2767-9764.CRC-25-0183

**Published:** 2025-08-22

**Authors:** Tina Akbarzadeh, Lin Ma, Jingyun Lee, Jade Moore, William Chou, Siyavash Shabani, Cristina M. Furdui, Bahram Parvin, Mary Helen Barcellos-Hoff

**Affiliations:** 1 Department of Radiation Oncology, Helen Diller Family Comprehensive Cancer Center, University of California, San Francisco, San Francisco, California.; 2 Department of Internal Medicine, Section on Molecular Medicine, Wake Forest University School of Medicine, Winston-Salem, North Carolina.; 3 Department of Electrical and Biomedical Engineering, College of Engineering, University of Nevada Reno, Reno, Nevada.

## Abstract

The risk of breast cancer increases decades after ionizing radiation exposure, thereby linking aging intrinsically to the evolution of cancer. We hypothesized that radiation accelerates aging and carcinogenesis through similar pathways, specifically low-grade systemic inflammation. In this study, we used the radiation-genetic mammary chimera model to examine the differential expression of 532 plasma proteins in BALB/c female mice between radiation exposure and experiment termination at 18 months. Mice were sham irradiated or irradiated with 50 cGy prior to being orthotopically transplanted with syngeneic *Trp53*-null mammary epithelium and half were treated for 6 months with anti-inflammatory low-dose aspirin. Plasma was collected at 4, 8, and 18 months from non–tumor-bearing mice and from those that had developed tumors between 12 and 18 months. Plasma quantitative proteomic analysis identified significant alterations in proteins involved in the inflammatory response in irradiated mice as a function of age. Levels of C4b-binding protein were decreased at 4 months in irradiated mice compared with controls, which was blocked in aspirin-treated irradiated mice. Notable differences in the expression of proteins associated with the inflammation were evident in tumor-bearing versus similarly aged mice. Complement components C1qA, C1qB, and C1qC were significantly increased in tumor-bearing mice that had been irradiated, whereas similarly aged mice without tumors displayed a decline in complement system activity. The specific changes in the complement system, which mediates adaptive immune function, following radiation exposure may contribute to cancer progression as a function of age.

**Significance:**

Women treated with radiotherapy as children or young adults bear an increased breast cancer risk, which is more likely to be aggressive, hormone receptor negative, and immune poor. Understanding radiation effects that could be modified after exposure may lead to prevention strategies. Consistent with our hypothesis that systemic inflammation contributes to risk, the plasma proteome from mice undergoing mammary carcinogenesis demonstrates changes in the complement system.

## Introduction

Ionizing radiation (IR) is a known carcinogen. The long-term health effects of radiation exposure documented in the Lifespan Study of atomic bomb survivors from Hiroshima and Nagasaki showed that a single, acute radiation dose increased the risk of breast cancer (1). Increased breast cancer risk is also observed for women treated with fractionated radiotherapy for Hodgkin lymphoma, whose risk is comparable with those with *BRCA1* germline mutations (2, 3). In both cases, there is a significant age-at-exposure effect in which the youngest women at exposure exhibit the highest rate of breast cancer later in life (4–6).

Breast cancer encompasses a diverse range of diseases, whose spectrum is primarily categorized by molecular characteristics. These include hormone receptor–positive cancers, which express estrogen and/or progesterone receptors, and HER2-positive breast cancer, marked by the overexpression of the HER2 protein, promoting aggressive cell growth. Less well appreciated is that radiotherapy-preceded breast cancers are more likely to be triple negative, i.e., lacking hormone receptors and amplification of HER2 (7). Radiotherapy-preceded breast cancers are also enriched for tumors that are devoid of lymphocytes (so-called immune-cold tumors) and have high levels of TGFβ and COX-2 (8). Although DNA mutations caused by radiation are believed to be the major carcinogenic mechanism, mutations cannot readily explain the difference in the breast cancer spectrum observed in radiotherapy-preceded breast cancers. We developed a radiation-mammary chimera model to determine other factors contribute to the unexpected difference in tumor types between sporadic and radiation-preceded breast cancers. This model separates radiation effects on the target mammary epithelium versus the host by using orthotopic transplantation of a *Trp53*-null epithelium, which gives rise to diverse mammary cancers, similar to the spectrum that occurs in women (9). This model shows that irradiating the host prior to transplantation is sufficient to shift the cancer spectrum from predominantly slow growing, hormone receptor–positive cancers to faster growing, hormone receptor–negative cancers (10). The transcriptomic profiles from tumors arising in irradiated hosts effectively clustered human basal-like cancers, supporting their commonalities (11). Notably, tumors lacking lymphocytes arose only in irradiated (IR) mice, and were also enriched in radiation-preceded breast cancer (8). Strikingly, 6 months of low-dose aspirin treatment before cancer development blocked the development of immunologically cold tumors, implicating systemic inflammation in the genesis of these cancers. Together, these data indicate that radiation effects on host biology are key to differences in the breast cancer spectrum.

The induction period between exposure and cancer development can span several decades in women and a year or more in the *Trp53*-null chimera model. Hence, carcinogenesis must be considered in the context of aging. A growing body of work posits that aging is a systemic process mediated by circulating factors that have immunosuppressive effects (12). The process of aging is linked to a decline in immune capabilities that contribute to heightened vulnerability to infections, cancer, and autoimmune disorders in older individuals. Inflammaging is the concept that chronic inflammation increases immunosuppressive immune cells that accelerate aging (12). Childhood cancer survivors treated with radiation have an earlier onset of age-related diseases (13), which raises the possibility that radiation accelerates aging.

We hypothesized that radiation accelerates aging and carcinogenesis through similar pathways, specifically low-grade systemic inflammation. To evaluate this idea, we collected plasma from BALB/c female mice irradiated with sham or 50 cGy prior to orthotopically transplanting with syngeneic *Trp53*-null mammary epithelium that were treated with or without low-dose aspirin for 6 months after transplantation. Plasma from the radiation-genetic mammary chimera model was collected at 4, 8, and 18 months from non–tumor-bearing mice and from those that had developed tumors between 12 and 18 months. Quantitative proteomics was used to identify 532 proteins and their differential expression as a function of age. Here, we report that proteins involved in the complement system were significantly altered as a function of carcinogenesis and age.

## Materials and Methods

### Mice

All animal experiments were performed at the University of California, San Francisco (UCSF). The protocols for animal husbandry and experiments were conducted with approval from the institutional review board and adhered to the NIH Guide for the Care and Use of Laboratory Animals. BABL/cJ *Mus musculus* (RRID: IMSF_JAX:000651) from The Jackson Laboratory were purchased and housed five per cage, fed LabDiet #5001 Rodent Formular (Purina Animal Nutrition LLC), and supplied with water *ad libitum*. BALB/c *Trp53* heterozygous mice were bred in-house to produce *Trp53*-null mice. *Trp53* status was confirmed through genotyping. At 9 weeks of age, the mammary glands were collected from *Trp53*-null female mice, diced into fragments, and viably frozen. All the studies were conducted at the UCSF Mt. Zion Cancer Center with the approval of the animal welfare committee. Mice were irradiated whole body with 50 cGy γ-rays using a ^137^Cs source irradiator at the UCSF Mt. Zion Cancer Center (8).

### Radiation-genetic mammary chimera model

**Figure 1:**
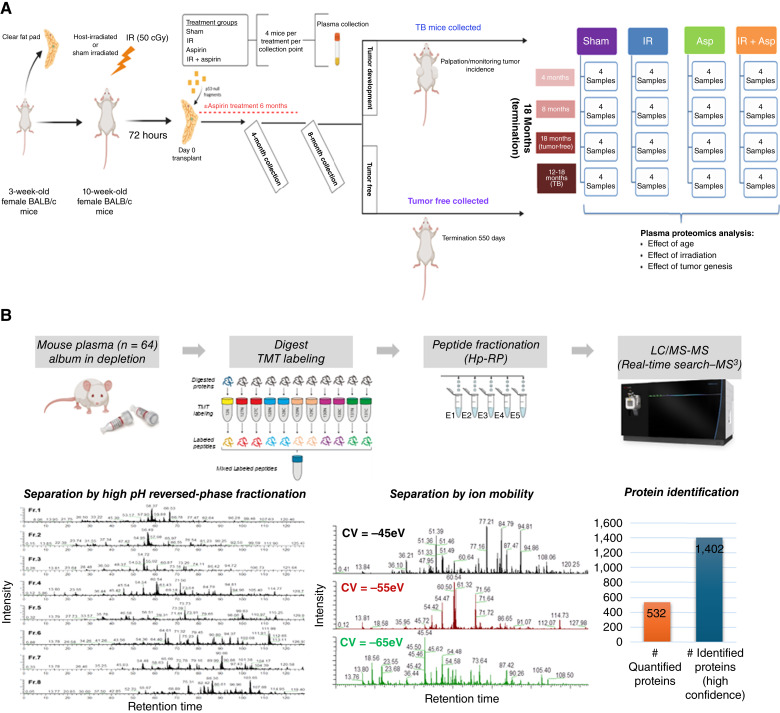
Experimental approach. **A,** The endogenous epithelium of the inguinal mammary glands was surgically removed from 3-week-old mice. These mice were then aged to 10 weeks, irradiated with 50 cGy of γ-ray, and transplanted with *Trp53*-null mammary fragments. Half of the mice received low-dose aspirin in their drinking water for 6 months. All mice were monitored by palpation, and subsets were euthanized at 4 and 8 months for plasma collection. The remaining mice were observed until tumor development or the study’s conclusion at 550 days after transplantation. Tissue samples were collected from mice that remained tumor-free up to 550 days of age. **B,** Graphical workflow of the proteomics. Asp, aspirin; Hp-RP, high pH reversed-phase.

The methodology and characterization of tumors from this experiment have been published (8). In brief, the epithelial bud was surgically excised from the inguinal mammary gland of 3-week-old BALB/c wild-type mice, which were implanted with *Trp53*-null fragments at 10 weeks of age to create the genetic chimera model (Fig. 1). Half of the mice were exposed to γ-radiation with a JL Shepherd Mark 1 Model-20 ^137^Cs source at a dose rate of 1.83 Gy/minute to a total body dose of 50 cGy 3 days before transplantation to create the radiation-genetic chimera. Half of each group received low-dose aspirin in their drinking water for 6 months after transplantation. For aspirin treatment, drinking water containing 0.1 mg/mL aspirin, refreshed weekly, was provided; as a mouse consumes approximately 4 mL water a day, the aspirin dose was approximately 0.4 mg/day. On the basis of body surface area conversion, this dose is similar to the human dose of 100 mg/day. Palpation was used to monitor all mice for tumor growth starting 1 month after transplantation. A randomly selected subset of each treatment group was euthanized at 4 and 8 months. The remaining mice were monitored until a tumor occurred between 12 and 18 months after transplantation. All remaining mice that did not develop a tumor were terminated at 550 days after transplantation, which is approximately 2 years of age.

Blood was collected at sacrifice from all mice by heart puncture into a K2-EDTA collection tube (BD Biosciences), gently mixed, and placed on ice. The specimen was transferred to a 1.7 mL siliconized tube (Bio Plas, Inc.) and centrifuged twice at 4°C and at 1,000 × *g* for 10 minutes each. The supernatant was aliquoted into three 1.7 mL siliconized tubes and stored at −80°C. Aliquots of plasma specimens were analyzed from four mice as a function of time after transplantation at 4, 8, and 18 months from each treatment group: control (sham irradiated), IR, aspirin treated, and IR mice treated with aspirin. Additional specimens from four tumor-bearing (TB) mice for each treatment were obtained at 12 to 18 months. The total number of specimens analyzed by quantitative proteomics was 64.

### Quantitative proteomic analysis of plasma

Plasma was processed through a Multiple Affinity Removal Spin Cartridge - Ms-3 (Mouse; Agilent Technologies, cat. #5188-5289) to deplete abundant proteins according to the manufacturer’s protocol. Briefly, plasma was diluted in buffer A (Agilent Technologies, cat. #5185-5987) and loaded onto the cartridge equilibrated with buffer A, and the flow-through was collected. The cartridge was washed using buffer A, and the eluent was combined with the primary flow-through, which was then further processed for proteomic analysis. The cartridge was cleaned by applying buffer B (Agilent Technologies, cat. #5185-5988) to remove the bound fraction for future use. Protein concentration in the collected flow-through was measured by bicinchoninic acid assay, and 100 μg protein was equally taken from every sample for tandem mass tagging (TMT) and relative quantification of plasma proteins across specimens by LC/MS-MS. Briefly, the 100-µg protein fraction was reduced with 10 mmol/L Tris (2-carboxy-ethyl)-phosphine-HCl and alkylated with 30 mmol/L iodoacetamide. The proteins were then precipitated by adding four times the sample volume of cold (−20°C) acetone, followed by incubation at −20°C overnight. Protein was isolated by centrifugation at 14,000 × *g* for 10 minutes. After removal of the supernatant, the protein pellet was air-dried at room temperature and resuspended in 100 mmol/L triethylammonium bicarbonate buffer, pH 8.5. Proteolysis was initiated by adding 2 μg of sequencing-grade modified trypsin and Lys-C mixture (Thermo Fisher Scientific, cat. #A41007) to the tube, followed by incubation at 37°C overnight. The reaction was stopped by lowering the pH with formic acid. The peptides were labeled using a TMT10plex Isobaric Label Reagents (Thermo Fisher Scientific, cat. #90111) according to the manufacturer’s protocol. To normalize the protein abundance across several multiplex mixtures, common reference samples were prepared by combining small aliquots from every sample prior to TMT labeling. The common reference sample was included in every TMT batch and labeled with TMT10-130C. Protein abundance belonging to the 130C quan-channel was used to normalize the corresponding protein abundance across all multiplex sets. TMT-labeled peptide mixture was fractioned using a High pH Reversed-Phase Peptide Fractionation Kit (Thermo Fisher Scientific, cat. #84868) according to the manufacturer’s protocol, resulting in eight fractions. Each fraction was dried and prepared in 5% (v/v) acetonitrile containing 0.1% (v/v) formic acid for LC/MS-MS analysis.

Peptides were analyzed on a Thermo Orbitrap Eclipse Mass Spectrometer coupled with a Vanquish Neo nano-UHPLC system (Thermo Fisher Scientific) through a high-field asymmetric waveform ion mobility spectrometry pro interface. Peptides were separated on a DNV PepMap Neo column (1,500 bar, 75 μm × 500 mm) using a 120-minute linear gradient of water (A) and 80% acetonitrile (B), both of which contained 0.1% formic acid. Data were collected using synchronous precursor selection-MS3–based TMT analysis and quantitation. MS2 spectra were acquired for peptide identification using a top-speed data-dependent scan, in which a maximum number of MS2 spectra were collected in 3 seconds per cycle between adjacent survey scans (MS1). MS3 scan was also performed for relative quantitation by multi-notch MS3-based TMT method in which synchronous precursor selection selected significant MS2 ions with the assistance of real-time database search which were fragmented to generate reporter ion peaks. These MS2 and MS3 scans were repeated on precursor ion subsets isolated by high-field asymmetric waveform ion mobility spectrometry, with compensation voltage set sequentially to –45 eV, –55 eV, and –65 eV. The dynamic exclusion option was enabled, which was set to 120 seconds. To identify proteins, spectra were searched against the UniProt mouse protein FASTA database (17,082 annotated entries, October 2021) using the Sequest HT search engine with the Proteome Discoverer v2.5 (Thermo Fisher Scientific, RRID: SCR_014477). Search parameters were as follows: FT-trap instrument; parent mass error tolerance, 10 parts per million; fragment mass error tolerance, 0.6 Da (monoisotopic); enzyme, trypsin (full); # maximum missed cleavages, two; variable modifications, +15.995 Da (oxidation) on methionine and +229.263 Da (TMT) on lysine and peptide N-term; and static modification, +57.021 Da (carbamidomethyl) on cysteine.

The results are reported herein as ratios of the abundance of each protein in a sample to its abundance in the “common reference” sample. These ratios identify relative differences in protein abundance between specimens, with higher or lower ratios indicating higher or lower protein levels compared with the standard reference across treatment conditions and time points (14). Commonly identified protein across specimens was expressed as the ratio of abundance averaged among the four specimens collected for each group relative to the “common reference” sample and then used to quantify changes in protein abundance between treatments. These data are provided in the supplementary Table S1.

### Statistical analysis

The data were analyzed using two-by-two comparisons, e.g., 4 months, irradiated to 4 months, control, to generate a list of proteins that displayed significant fold change and *P* value < 0.05 based on a Student *t* test. Unsupervised clustering was performed using bidirectional hierarchical clustering to simultaneously group both rows and columns of the dataset. The analysis was based on Euclidean distance and the Ward.D2 linkage method to assess similarity and construct the clustering dendrograms. Some results are visually represented in a volcano plot, which plots the negative 10 logarithms of the *P* value against two logarithms of the fold change for each protein. These data were subjected to network analysis to map proteins onto known biological pathways (15–18).

The normality of the data distribution was assessed using the Shapiro–Wilk test. Data met the assumption of normality and were suitable for parametric testing. A two-way ANOVA was performed using GraphPad Prism (GraphPad Software 10.3.1, RRID: SCR_002798) to evaluate the effects of IR and aspirin treatment or the combination on relative plasma protein abundance, which was calculated as the ratio of each sample’s intensity to the corresponding reference sample in the same batch. These normalized, unitless values reflect relative abundance and were used for downstream statistical analysis. As the data were already normalized to control batch effects and ensure comparability, no additional transformation was applied. One specimen from the 18-month time point, sham-treated and TB group was excluded from analysis because of missing or undetectable proteomic data.

### Data availability

The mass spectrometry proteomics data generated in this study have been deposited to the ProteomeXchange Consortium via the PRIDE partner repository (RRID: SCR_012052). The data are available via ProteomeXchange with identifier PXD064950.

## Results

### Plasma proteome of female BALB/c mice

**Figure 2:**
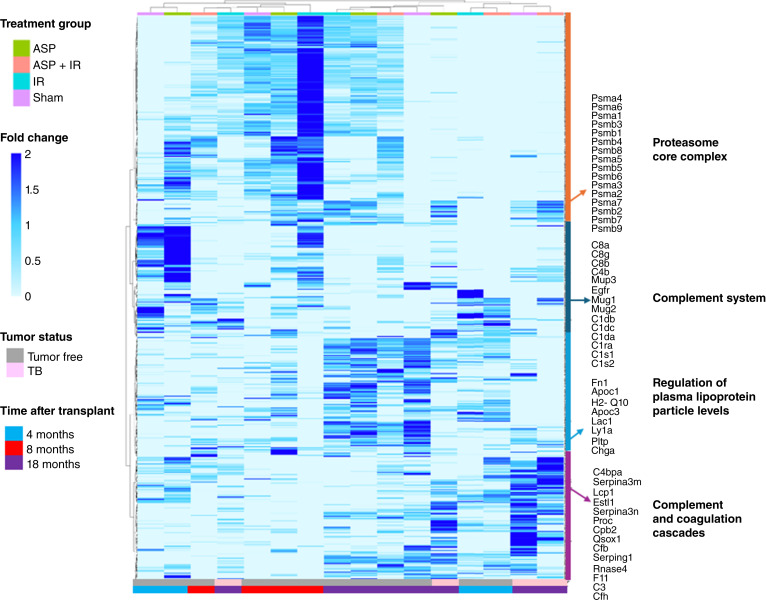
Differential plasma protein abundance as a function of treatment and time. Unsupervised clustering of plasma protein abundance annotated for treatment group [control, aspirin (ASP), IR, and IR + aspirin] and time of post-transplantation (4, 8, and 18 months) of tumor free and TB mice. The key pathway enrichment of proteins in the four clades is indicated in the sidebar. Unsupervised clustering using Euclidian distance and Ward.D.2.

The abundance of 532 detected proteins was measured in plasma as a function of time after transplantation (i.e., age), treatment group, and the presence of a mammary tumor. Supervised heatmaps display differences averaged among proteins from tumor-free and TB mice (*n* = 4) from a given treatment (Supplementary Fig. S1) and age group (Supplementary Fig. S2). However, as both age and the carcinogenic process are time-varying, we focused on unsupervised clustering, which revealed four major clades (Fig. 2). From the top, clade 1 was characterized by the proteasome core complex. The second clade was enriched in proteins involved in the complement system. The third was characterized by lipoprotein particle levels, particularly in mice aged 18 months after transplantation. The fourth was enriched in complement and coagulation cascades, particularly in TB mice.

Notably, the major variable associated with each clade was age rather than treatment. Clade 1 clustered specimens 8 months after transplantation, clade 2 clustered those obtained at 4 months, and clade 3 clustered specimens from 18 months. Clade 4 was associated with TB mice.

A total of 67 proteins were increased or decreased by the transition from young to middle-aged in sham-treated (control) mice. These included the complement C1s-B subcomponent, C4b-binding protein (C4BP), complement C1r-A subcomponent, and complement C1q subcomponent subunit A (C1QA), which were significantly decreased by aging.

In aspirin-treated mice, half as many (*n* = 32) proteins changed with increased age. Some of these proteins, which belong to the lipoprotein pathways, are significantly increased with age, such as apolipoprotein C-I, CIII, and C-IV. This increase is consistent with the effect of aspirin on lipid metabolism (19).

A total of 197 proteins changed in IR mice compared with controls. Most proteins decreased at 8 months compared with 4 months (162 proteins decreased and 35 proteins increased). As in control mice, aspirin treatment in IR mice more than halved the number of significantly changed proteins to only 70 proteins. Those proteins included coagulation factor XI, XII, and XIII B chain, which significantly decreased at 8 months. Significantly increased proteins belonged to the protein-disulfide reductase [NAD(P)] activity pathway, like thioredoxin and glutaredoxin-1.

Comparison of the protein expression at 8 versus 18 months also revealed variations as a function of treatment: 81 proteins were significantly different in control mice, and 96 proteins were different in IR mice, whereas those treated with aspirin had 70 proteins and only 52 proteins differed in those IR mice treated with aspirin. Interestingly, regardless of the treatment, our analysis revealed consistent enrichment in the complement and coagulation cascades and the renin–angiotensin system between these two time points. Proteins such as C4BP and vitronectin significantly increased with aging.

### Proteome of IR mice

**Figure 3:**
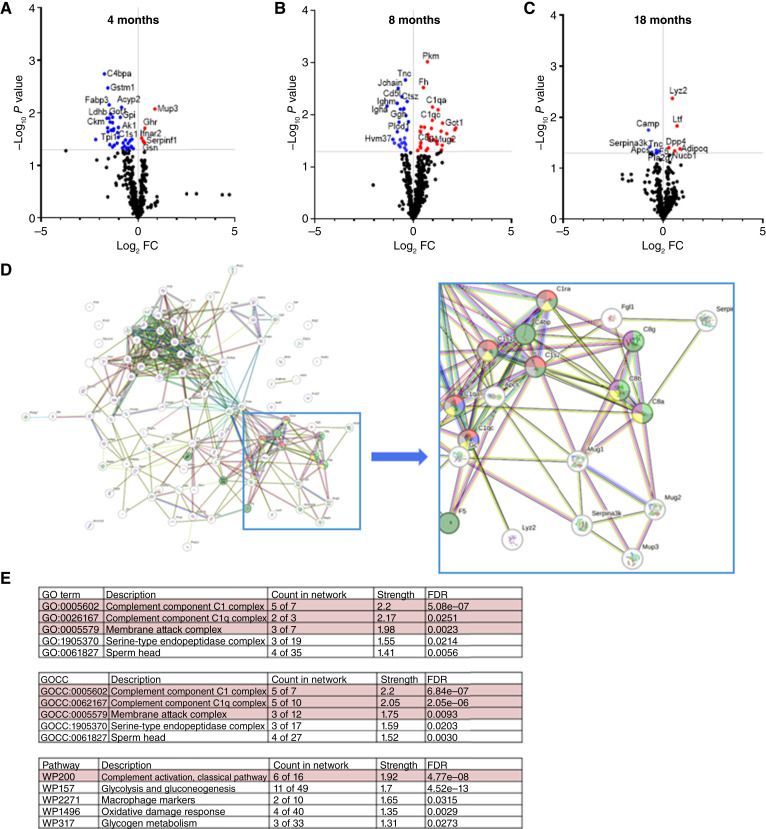
Network analysis of differentially expressed proteins in irradiated mice. **A–C,** Volcano plot of differentially expressed proteins in tumor-free mice from the IR group compared with controls at (**A**) 4 months, (**B**) 8 months, and (**C**) 18 months. **D,** Network analysis highlights the complement system as a predominant pathway. **E,** Pathway enrichment of complement systems indicated by gene ontology, components, and WIKI Pathways. Each colored circle in the table represents the placement of a protein within the network. Proteins that occur in multiple colors in the network indicate their simultaneous involvement in multiple highlighted pathways.

Although age is the major factor associated with protein clusters, we were interested in whether radiation subtly perturbed this. By comparing differentially expressed proteins in plasma from IR mice with controls at each time point, we identified changes as a function of time after irradiation (Fig. 3A–C). These proteins were subjected to network analysis to map proteins onto known biological pathways (15–18). Network analysis (Fig. 3D) and pathway enrichment (Fig. 3E) identified the complement system as one of the most enriched pathways. C4BP is critical in regulating the complement system. Here, a significant reduction in C4BP levels was observed in the IR group at the 4-month collection time. This decrease suggests impairment or alteration in the ability to regulate the complement cascade after irradiation. Such dysregulation can lead to increased vulnerability to infections and a greater propensity for inflammatory responses, which are common complications in irradiated tissues (20). The reduction in C4BP could enhance complement activation, leading to increased tissue damage and influencing the overall pathophysiologic response to irradiation.

### Proteome as a function of time and treatment

**Figure 4:**
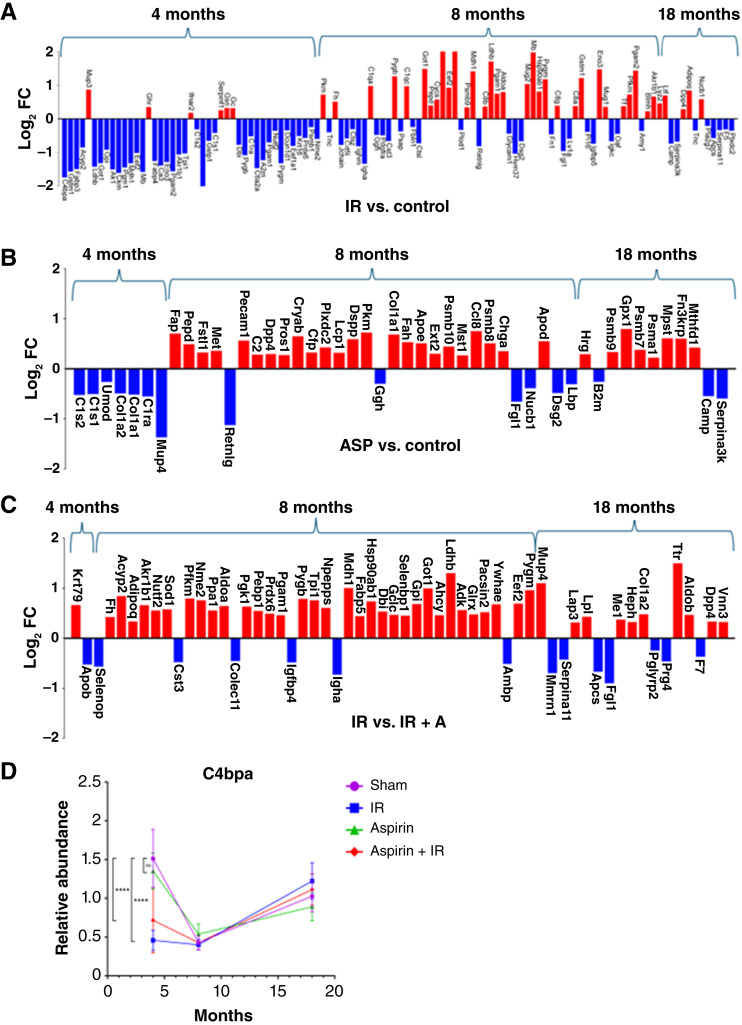
Temporal expression of proteins as a function of treatment. **A–C,** Significantly differentially expressed proteins (Student *t* test) as a function of time and treatment. **D,** C4b-binding protein levels across time for each treatment (sham, purple; IR, turquoise; aspirin, green; and IR + aspirin, pink). Error bars represent SE, and asterisks denote statistical significance determined by a Student *t* test. ASP, aspirin.

The expression of plasma proteins differed as a function of treatment and time after irradiation (Fig. 4). Most plasma proteins were decreased in IR mice compared with controls at 4 months, but by 8 months, the majority of differentially expressed proteins were increased, whereas by 18 months, the few proteins that were differentially expressed were distributed between increased and decreased (Fig. 4A). As noted above, mice treated with aspirin had decreased expression of seven proteins, but by 8 months, most proteins were increased compared with controls. Fewer proteins were affected at 18 months, but the majority were increased (Fig. 4B). Aspirin treatment of IR mice eliminated almost all the protein increases at 4 months and decreased the magnitude and composition of changes at 8 months; by 18 months, these mice had a similar number of plasma protein changes as IR mice, but different proteins were differentially expressed (Fig. 4C). These complex patterns are exemplified by C4BP expression (Fig. 4D). IR mice had decreased C4BP at the early time point, independent of aspirin treatment, but it did not persist at later collection times of 8 and 18 months, suggestive of an acute phase, transient response specific to the post-irradiation period.

### Proteome of cancer-bearing mice

**Figure 5:**
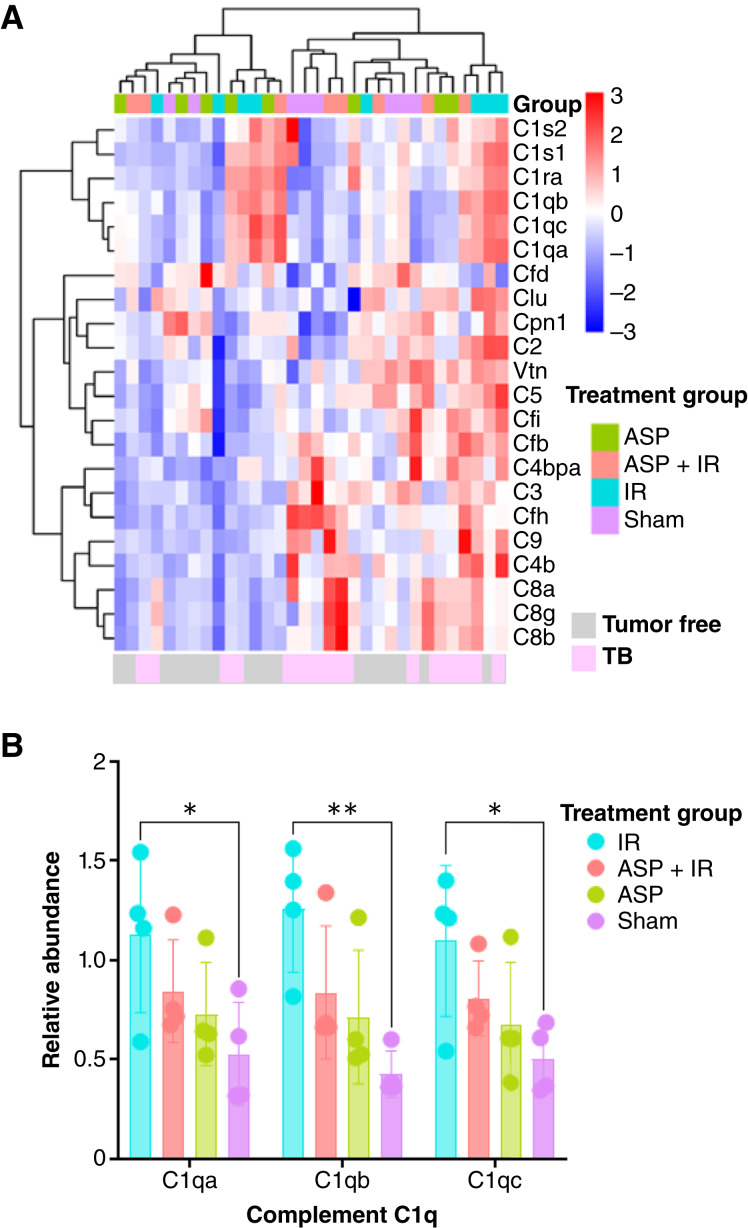
Complement system proteins of plasma from TB mice compared with similarly aged non-TB mice. **A,** Heatmap representing unsupervised clustering of complement system components in TB mice (red bottom bar) vs. tumor-free mice at termination (blue bottom bar). Unsupervised clustering using Euclidian distance and Ward.D.2. **B,** Differential expression of complement C1q family members, a, b, and c, in TB treated as indicated. Bars represent means (*n* = 4) and SE. Statistical significance between treatment groups for each complement component defined by a two-way ANOVA is denoted by asterisks above the bars. *, *P* < 0.05; **, *P* < 0.01.

Next, we compared the plasma proteins of TB mice that were 12 to 18 months old with those that were 18 months old and tumor-free at the time of termination (Fig. 5A). A total of 36 proteins were increased or decreased in TB mice compared with similarly aged controls. Complement system components such as complement factor H, C4BP, and collectin-11 were significantly increased in TB mice, which was the opposite of what occurs with aging alone. In the TB mice previously treated with aspirin, 55 proteins were changed compared with similarly aged non-TB mice, including significantly increased proteins belonging to the lipoprotein pathways, such as apolipoprotein M, which may reflect increased lipid metabolism due to aspirin therapy.

Complement system components such as complement factor H and complement component C8 α, β, and γ chain were significantly increased in TB mice. In the TB radiation-chimera mice, 26 proteins were changed significantly. Notably, all these proteins were decreased and are involved in response-to-stimulus pathways. In the TB IR + aspirin-treated mice, 61 proteins significantly changed; most were decreased (54 proteins decreased and seven proteins increased). Of note, the seven increased proteins were C-reactive protein, proteasome subunit β type-8, serine protease inhibitor A3K, C–C motif chemokine 8, prosaposin, complement C3, and α-1-antitrypsin 1 to 4.

The complement system, particularly components C1qA, C1qB, and C1qC, plays critical roles in the body’s innate immune defenses. Notably, TB mice that had been irradiated had significantly elevated C1qA, C1qB, and C1qC levels, which were eliminated by aspirin treatment (Fig. 5B). Together, these data support that low-dose radiation elicits systemic inflammation that may contribute to both aging and carcinogenesis.

## Discussion

This study aimed to identify plasma proteins significantly altered by prior radiation exposure across the lifespan and those altered by the development of cancer. By analyzing each treatment group as a function of age, we sought to isolate the specific protein changes related to treatment from the presence of tumors within similarly aged cohorts. We identified consistent changes in complement proteins in the plasma associated with both radiation exposure and cancer development.

Complement system effects on innate immune regulation and cancer are complex, and the mechanisms and molecules that activate the complement cascade during carcinogenesis are largely unknown (21). The complement system is comprised of membrane-associated proteins and small proteins found in blood plasma that circulate in inactive forms, which, upon activation, result in a sequential cascade of enzymatic reactions. Activation of the complement system can occur through three main pathways: the classical pathway, the lectin pathway, and the alternative pathway (22). Activation leads to a series of responses, including opsonization of pathogens, recruitment of inflammatory cells, and formation of the membrane attack complex that can lyse pathogen cells (23). Complement was initially thought to play an essential role in innate immunity, in which an intense and rapid response is mounted against invading pathogens (24, 25). However, it has recently become increasingly apparent that complement also plays a vital role in adaptive immunity involving T and B cells that help eliminate pathogens (24). Hence, complement is involved in innate and adaptive immunity, mediating tissue regeneration, tumor growth, and other pathologic conditions (22, 26).

The role of the complement system in cancer is a target for therapeutic intervention, but is still poorly understood because it plays a dual role, with evidence of its contribution to tumor promotion and suppression (27, 28). Specific components of the complement system can contribute to tumor growth by creating a chronic inflammatory environment that promotes cancer progression (29). On the other hand, complement activation regulates the adaptive immune response and may play a role in regulating the T-cell control of tumors (28).

The distinct difference between proteins that mediate inflammation in TB and aged mice emphasizes the adaptive changes in the immune system under different biological stresses. During carcinogenesis, the immune system is activated but incapacitated locally by the tumor (30). In aging, the deterioration of the immune system is reflected by the reduced activity of the complement system, characterized by impaired immune cell function and chronic inflammation by as yet poorly defined mechanisms. A more detailed understanding of these distinct roles is critical in understanding how the immune system, which is evolving with age, is affected by cancer, insights that could be critical to developing age-specific or cancer-specific interventions.

Previous comprehensive plasma proteomic analysis identified conserved aging patterns for 46 plasma proteins between humans and mice (31). Five of these proteins, Cast, Igf1, Mb, Mrc1, and Stip1, were identified in our study. Stip1 and Cast followed the general pattern observed in humans of increasing from early to middle age, but were not increased in aged (18 month) post-transplantation female mice. In contrast, insulin-like growth factor 1, which decreases with age in humans, seemed to be most associated with aspirin treatment in our study and was significantly increased in TB mice treated with aspirin compared with aged mice. Interestingly, Mrc1, which increases with age in humans, decreased in TB mice compared with aged mice.

C4BP regulates the complement system, especially in the classical and lectin pathways. It acts mainly as a control protein, preventing the formation and activity of C3/C5 convertases and causing the decay of already formed convertase complexes. This regulatory action is critical in avoiding uncontrolled activation of the complement system, which can lead to inflammation and tissue damage. C4BP also binds protein S, a cofactor of activated protein C, which is involved in the inactivation of factors Va and VIIIa in the coagulation cascade, thereby linking the complement system to coagulation pathways and inflammatory responses (32). In addition, C4BPA has been detected in tissue and blood samples taken from patients with breast cancer (33).

The consequences of decreased C4BP in IR mice at the 4-month mark could extend beyond direct effects on complement regulation. Given the role of C4BP in linking the complement system to coagulation, its reduction may also affect homeostatic processes. This can lead to an increased risk of bleeding or thrombotic events. Understanding these interactions and the molecular mechanisms underlying the decrease of C4BP after radiation can help reduce the adverse effects of radiation. Further research on the pathways affected by changes in C4BP levels could provide deeper insights into the systemic effects of radiation and suggest potential biomarkers or therapeutic targets. The transient nature of C4BP reduction at the 4-month collection time highlights the dynamic responses of the immune system to radiation and emphasizes the need for time-specific interventions in therapeutic strategies.

Also, the complement system, especially the components C1qA, C1qB, and C1qC, plays a vital role in the body’s innate immune defense. Together, these components form the recognition unit of the C1 complex. C1q is a critical component of the classical complement activation pathway and a key link between innate immunity mediated by the classical pathway and acquired immunity mediated by IgG or IgM (34). It is essential to understand the modulation of these components in different physiologic and pathologic contexts.

Our experiment identified changes in the complement components in TB, IR mice versus TB controls in that plasma C1qA, C1qB, and C1qC levels significantly increased in IR mice. This upregulation of the classical pathway suggests that early radiation exposure activates the immune system, which might help recognize and respond to neoplastic cells. For example, an increase in these complement components can lead to a more pronounced opsonization process and subsequent phagocytosis of tumor cells that enhances immune-mediated tumor cell clearance (35). The persistent complement activity in the context of irradiated TB mice could be evidence of an active immune response against neoplastic cells or could increase the risk of peripheral tissue damage or autoimmune phenomena due to the nonspecific nature of complement-mediated cell lysis and inflammation (36).

Further studies are necessary to elucidate the precise mechanisms by which low-dose radiation affects the expression and activity of complement components C1qA, C1qB, and C1qC in TB mice. The interplay between radiation exposure, carcinogenesis, and aging is undoubtedly complex but may be tractable by focusing efforts on deciphering their respective effects on the immune system.

## Supplementary Material

Supplementary Figure S1 Figure S1. Supervised heatmaps of differentially abundant plasma proteins across treatment groups.

Supplementary Figure S2 Figure S2. Supervised heatmap of differentially abundant plasma proteins across time points.

Supplementary Table 1 Table S1

## References

[1] Ozasa K , Shimizu Y, Suyama A, Kasagi F, Soda M, Grant EJ, . Studies of the mortality of atomic bomb survivors, Report 14, 1950–2003: an overview of cancer and noncancer diseases. Radiat Res 2012;177:229–43. 22171960 10.1667/rr2629.1

[2] Bhatia S , Robison LL, Oberlin O, Greenberg M, Bunin G, Fossati-Bellani F, . Breast cancer and other second neoplasms after childhood Hodgkin’s disease. N Engl J Med 1996;334:745–51. 8592547 10.1056/NEJM199603213341201

[3] Bhatia S , Yasui Y, Robison LL, Birch JM, Bogue MK, Diller L, . High risk of subsequent neoplasms continues with extended follow-up of childhood Hodgkin’s disease: report from the Late Effects Study Group. J Clin Oncol 2003;21:4386–94. 14645429 10.1200/JCO.2003.11.059

[4] Ronckers CM , Erdmann CA, Land CE. Radiation and breast cancer: a review of current evidence. Breast Cancer Res 2005;7:21–32. 15642178 10.1186/bcr970 PMC1064116

[5] Koo E , Henderson MA, Dwyer M, Skandarajah AR. Radiation-associated breast cancers in a late-effects cohort: long-term surveillance is essential. Asia Pac J Clin Oncol 2020;16:363–71. 32894009 10.1111/ajco.13382

[6] Sauder CAM , Li Q, Othieno A, Cruz D, Arora M, Bold RJ, . Characteristics and outcomes for secondary breast cancer in childhood, adolescent, and young adult cancer survivors treated with radiation. Cancer Epidemiol Biomarkers Prev 2020;29:1767–74. 32847936 10.1158/1055-9965.EPI-20-0260 PMC9377811

[7] Horst KC , Hancock SL, Ognibene G, Chen C, Advani RH, Rosenberg SA, . Histologic subtypes of breast cancer following radiotherapy for Hodgkin lymphoma. Ann Oncol 2014;25:848–51. 24608191 10.1093/annonc/mdu017

[8] Ma L , Gonzalez-Junca A, Zheng Y, Ouyang H, Illa-Bochaca I, Horst KC, . Inflammation mediates the development of aggressive breast cancer following radiotherapy. Clin Cancer Res 2021;27:1778–91. 33402361 10.1158/1078-0432.CCR-20-3215 PMC7956216

[9] Nguyen DH , Ouyang H, Mao J-H, Hlatky L, Barcellos-Hoff MH. Distinct luminal type mammary carcinomas arise from orthotopic Trp53 null mammary transplantation of juvenile versus adult mice. Cancer Res 2014;74:7149–58. 25281718 10.1158/0008-5472.CAN-14-1440 PMC4252877

[10] Nguyen DH , Oketch-Rabah HA, Illa-Bochaca I, Geyer FC, Reis-Filho JS, Mao J-H, . Radiation acts on the microenvironment to affect breast carcinogenesis by distinct mechanisms that decrease cancer latency and affect tumor type. Cancer Cell 2011;19:640–51. 21575864 10.1016/j.ccr.2011.03.011 PMC3110779

[11] Nguyen DH , Fredlund E, Zhao W, Perou CM, Balmain A, Mao J-H, . Murine microenvironment metaprofiles associate with human cancer etiology and intrinsic subtypes. Clin Cancer Res 2013;19:1353–62. 23339125 10.1158/1078-0432.CCR-12-3554 PMC3732211

[12] Leonardi GC , Accardi G, Monastero R, Nicoletti F, Libra M. Ageing: from inflammation to cancer. Immun Ageing 2018;15:1. 29387133 10.1186/s12979-017-0112-5 PMC5775596

[13] Yeh JM , Ward ZJ, Stratton KL, McMahon MV, Taylor CS, Armstrong GT, . Accelerated aging in survivors of childhood cancer-early onset and excess risk of chronic conditions. JAMA Oncol 2025;11:535–43. 40111318 10.1001/jamaoncol.2025.0236 PMC11926734

[14] Michiels TJM , van Veen MA, Meiring HD, Jiskoot W, Kersten GFA, Metz B. Common reference-based tandem mass Tag multiplexing for the relative quantification of peptides: design and application to degradome analysis of diphtheria toxoid. J Am Soc Mass Spectrom 2021;32:1490–7. 33983728 10.1021/jasms.1c00070 PMC8176455

[15] Ashburner M , Ball CA, Blake JA, Botstein D, Butler H, Cherry JM, . Gene ontology: tool for the unification of biology. The Gene Ontology Consortium. Nat Genet 2000;25:25–9. 10802651 10.1038/75556 PMC3037419

[16] Aleksander SA , Balhoff J, Carbon S, Cherry JM, Drabkin HJ, Ebert D, ; Gene Ontology Consortium. The Gene Ontology knowledgebase in 2023. Genetics 2023;224:iyad031. 36866529 10.1093/genetics/iyad031 PMC10158837

[17] Agrawal A , Balcı H, Hanspers K, Coort SL, Martens M, Slenter DN, . WikiPathways 2024: next generation pathway database. Nucleic Acids Res 2024;52:D679–89. 37941138 10.1093/nar/gkad960 PMC10767877

[18] Szklarczyk D , Kirsch R, Koutrouli M, Nastou K, Mehryary F, Hachilif R, . The STRING database in 2023: protein–protein association networks and functional enrichment analyses for any sequenced genome of interest. Nucleic Acids Res 2023;51:D638–46. 36370105 10.1093/nar/gkac1000 PMC9825434

[19] Lu X-R , Liu X-W, Li S-H, Qin Z, Bai L-X, Ge W-B, . Untargeted lipidomics and metagenomics reveal the mechanism of aspirin eugenol ester relieving hyperlipidemia in ApoE−/− mice. Front Nutr 2022;9:1030528. 36618709 10.3389/fnut.2022.1030528 PMC9815714

[20] Lumniczky K , Impens N, Armengol G, Candéias S, Georgakilas AG, Hornhardt S, . Low dose ionizing radiation effects on the immune system. Environ Int 2021;149:106212. 33293042 10.1016/j.envint.2020.106212 PMC8784945

[21] Zhang B , Whiteaker JR, Hoofnagle AN, Baird GS, Rodland KD, Paulovich AG. Clinical potential of mass spectrometry-based proteogenomics. Nat Rev Clin Oncol 2019;16:256–68. 30487530 10.1038/s41571-018-0135-7 PMC6448780

[22] Mastellos DC , Hajishengallis G, Lambris JD. A guide to complement biology, pathology and therapeutic opportunity. Nat Rev Immunol 2024;24:118–41. 37670180 10.1038/s41577-023-00926-1

[23] Sarma JV , Ward PA. The complement system. Cell Tissue Res 2011;343:227–35. 20838815 10.1007/s00441-010-1034-0 PMC3097465

[24] Pouw RB , Ricklin D. Tipping the balance: intricate roles of the complement system in disease and therapy. Semin Immunopathol 2021;43:757–71. 34698894 10.1007/s00281-021-00892-7 PMC8547127

[25] Merle NS , Noe R, Halbwachs-Mecarelli L, Fremeaux-Bacchi V, Roumenina LT. Complement system part II: role in immunity. Front Immunol 2015;6:257. 26074922 10.3389/fimmu.2015.00257 PMC4443744

[26] Ong J , Zarnegar A, Selvam A, Driban M, Chhablani J. The complement system as a therapeutic target in retinal disease. Medicina (Kaunas) 2024;60:945. 38929562 10.3390/medicina60060945 PMC11205777

[27] Lu P , Ma Y, Wei S, Liang X. The dual role of complement in cancers, from destroying tumors to promoting tumor development. Cytokine 2021;143:155522. 33849765 10.1016/j.cyto.2021.155522

[28] Afshar-Kharghan V . The role of the complement system in cancer. J Clin Invest 2017;127:780–9. 28248200 10.1172/JCI90962 PMC5330758

[29] Pajares MJ , Agorreta J, Salvo E, Behrens C, Wistuba II, Montuenga LM, . TGFBI expression is an independent predictor of survival in adjuvant-treated lung squamous cell carcinoma patients. Br J Cancer 2014;110:1545–51. 24481402 10.1038/bjc.2014.33 PMC3960613

[30] Mittal D , Gubin MM, Schreiber RD, Smyth MJ. New insights into cancer immunoediting and its three component phases-elimination, equilibrium and escape. Curr Opin Immunol 2014;27C:16–25. 10.1016/j.coi.2014.01.004 PMC4388310 24531241

[31] Lehallier B , Gate D, Schaum N, Nanasi T, Lee SE, Yousef H, . Undulating changes in human plasma proteome profiles across the lifespan. Nat Med 2019;25:1843–50. 31806903 10.1038/s41591-019-0673-2 PMC7062043

[32] Rezende SM , Simmonds RE, Lane DA. Coagulation, inflammation, and apoptosis: different roles for protein S and the protein S–C4b binding protein complex. Blood 2004;103:1192–201. 12907438 10.1182/blood-2003-05-1551

[33] Zou J , Chen Y, Ji Z, Liu D, Chen X, Chen M, . Identification of C4BPA as biomarker associated with immune infiltration and prognosis in breast cancer. Transl Cancer Res 2024;13:25–45. 38410217 10.21037/tcr-23-1215 PMC10894332

[34] Chen L-H , Liu J-F, Lu Y, He X-Y, Zhang C, Zhou H-H. Complement C1q (C1qA, C1qB, and C1qC) may be a potential prognostic factor and an index of tumor microenvironment remodeling in osteosarcoma. Front Oncol 2021;11:642144. 34079754 10.3389/fonc.2021.642144 PMC8166322

[35] Gao C , Kozlowska A, Nechaev S, Li H, Zhang Q, Hossain DMS, . TLR9 signaling in the tumor microenvironment initiates cancer recurrence after radiotherapy. Cancer Res 2013;73:7211–21. 24154870 10.1158/0008-5472.CAN-13-1314 PMC3917779

[36] Galindo-Izquierdo M , Pablos Alvarez JL. Complement as a therapeutic target in systemic autoimmune diseases. Cells 2021;10:148. 33451011 10.3390/cells10010148 PMC7828564

