# Supplementary material for: Plasma Proteomic Analysis Reveals Complement System Changes in Irradiated Female BALB/c Mice during Mammary Carcinogenesis
Source: Cancer Res Commun. 2025 Aug 22;5(8):1409–18. doi: 10.1158/2767-9764.CRC-25-0183 (PMC12371320; doi:10.1158/2767-9764.CRC-25-0183)

**Supplementary Figure S1.** Supervised heatmaps of differentially abundant plasma proteins across treatment groups. (Top bar: Sham, purple; IR, turquoise; Aspirin, green; Aspirin+IR, coral pink). Columns represent the average protein abundance for each treatment group at each collection time point. Rows represent individual proteins. (Bottom bars: tumor free, gray; tumor bearing, pink; 4 months, blue; 8 months, red; 18 months, dark purple)


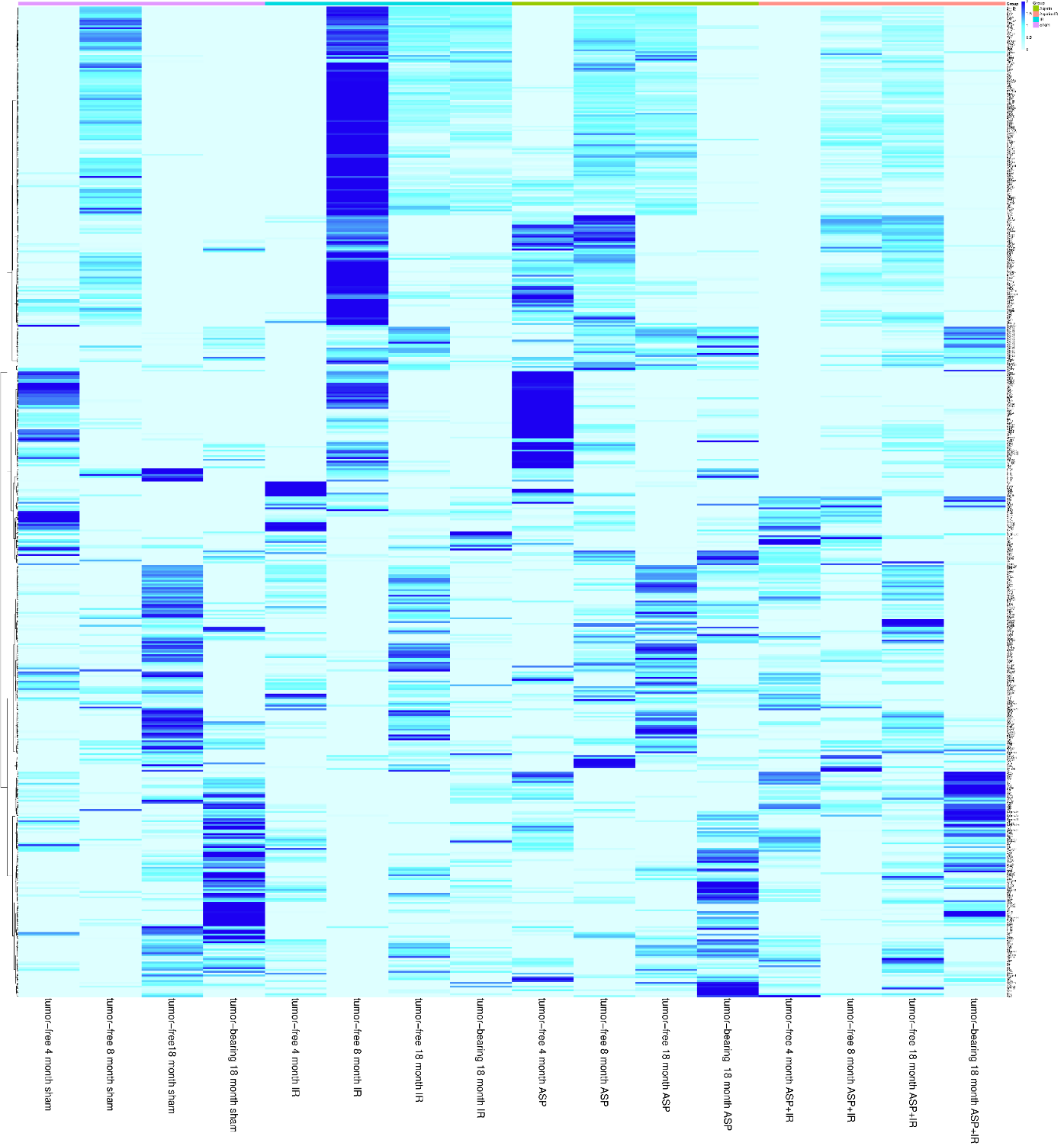

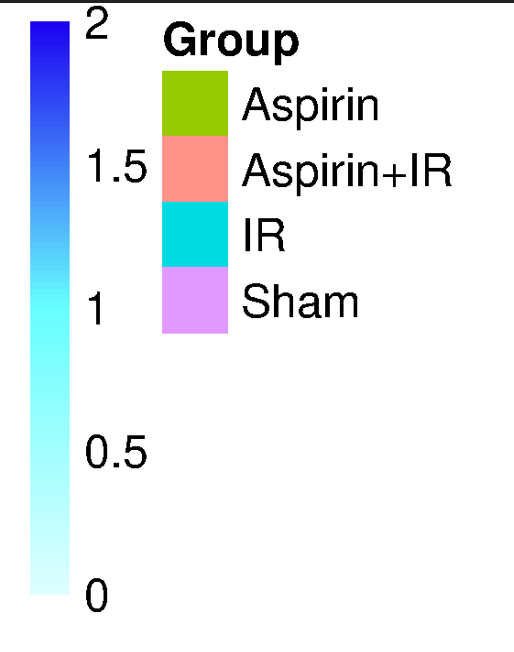

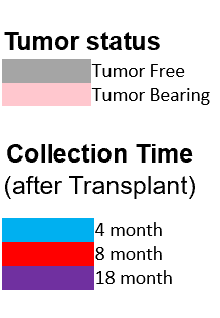

Supplement: Supplementary Figure S1 — Figure S1. Supervised heatmaps of differentially abundant plasma proteins across treatment groups. [file crc-25-0183_supplementary_figure_s1_suppsf1.docx]
